# Physico-chemical oxidative cleavage strategy facilitates the degradation of recalcitrant crystalline cellulose by cellulases hydrolysis

**DOI:** 10.1186/s13068-018-1016-0

**Published:** 2018-01-25

**Authors:** Huan Zhou, Liuyang Wang, Yun Liu

**Affiliations:** Beijing Key Laboratory of Bioprocess, College of Life Science and Technology, Beijing University of Chemical Technology, Beijing, 100029 China

**Keywords:** Crystalline cellulose, Irradiation oxidation, Post-reduction, Enzymatic hydrolysis, Degradation mechanism

## Abstract

**Background:**

Efficient enzymatic conversion of recalcitrant crystalline cellulose is critical for enabling cost-effective industrial conversion of cellulosic biomass to biofuels and chemicals. Fully understanding enzyme digestion mechanism is paving a new way to design efficient process for biomass conversion. Accordingly, a continuing drive is inspiring to discover new routes to promote crystalline cellulose disruption.

**Results:**

Herein, a physico-chemical oxidative cleavage strategy of irradiation oxidation/post-reduction (IOPR) was employed to treat crystalline cellulose I to cleave glycosidic bonds association with some new oxidized and reduced chain ends, thus boosting downstream degradation by cellulases from *Trichoderma reesei*. The hydrolysis performance of treated crystalline cellulose was conducted with either *T. reesei* Cel7A (TrCel7A) alone, or a cellulase enzyme mixture (90% Celluclast 1.5 L, 10% β-glucosidase). 81.6 and/or 97% of conversion efficiency have been reached for 24-h and 48-h cellulase hydrolysis, respectively. The high efficient conversion of crystalline cellulose after IOPR is mainly attributed to generating some new chain ends, which are identified by MAIDI-TOF–MS and HPLC. Furthermore, the nanoscale architectures of crystalline cellulose before and after IOPR are systematically investigated by XRD, EPR, ATR- FTIR, GPC, and XPS techniques. Together with TEM images, the results reveal a fascinating digestion mechanism of “peel-off” and “cavity-formation” paradigms toward degrading crystalline cellulose by cellulase mixtures after IOPR treatment.

**Conclusions:**

This encouraging results show that the proposed IOPR approach will become a potential competitive alternative to current biomass pretreatment. It opens a new avenue toward the implementation of pretreatment and the design of enzyme cocktails in lignocellulosic biorefinery.

**Electronic supplementary material:**

The online version of this article (10.1186/s13068-018-1016-0) contains supplementary material, which is available to authorized users.

## Background

Cellulose, the most abundant polysaccharides with a linear chain polymer of β-1,4-linked d-glucose units in nature, is playing a pivotal role in the enzyme-based “biorefinery” from lignocellulose [1]. As an ideal alternative of petroleum-based source, cellulose can be hydrolyzed into monomeric sugars for downstream conversion to a wide range of fuels, chemicals, and materials; thus it will eliminate the associated economic, environmental, and energy security issues of fossil-based fuels production [2]. However, the remarkable recalcitrance of crystalline cellulose has hitherto held one limiting step in this enzyme-based “biorefinery” process [3].

Numerous approaches have been developed to accelerate enzymatic hydrolysis of crystalline cellulose into glucose, including dilute acid/alkali, ammonia fiber expansion (AFEX), ionic liquids, organosolv methods [4–7]. These above techniques have demonstrated two major effects, one is to increase the enzymes accessibility to binding cellulose surface; the other is to decrease the free energy of enzymes for processive decrystallization through reducing cellulose crystallinity [8, 9]. However, complexities associated with both the cost-effective industrialization and large amount of chemicals consumption are still the major challenges for practical utilization of these approaches.

In nature, biomass-degrading microorganisms have evolved a series of enzymes to help digest crystalline cellulose. For example, the well-characterized brown rot fungi have a more efficient cellulolytic system depending on an oxidative–hydrolytic two-step mechanism [10], it was found that lignocellulose was firstly oxidized by reactive oxygen species (ROS) from enzymes prior to hydrolysis by glycoside hydrolases [11]. Recently, lytic polysaccharide monooxygenases (LPMOs) have shown the synergistical interaction with hydrolytic cellulases to break down crystalline cellulose by an oxidative cleavage mechanism [12–14]. It was demonstrated that LPMOs not only introduce oxidized chain breaks in the polysaccharide chains, but also decrease the free energy for decrystallization [15, 16]. These unexpected findings provide new perspectives to design more efficient biorefinery process in future.

In our previous works, *γ*-ray irradiation pretreatment could facilitate enzymatic hydrolysis of crystalline cellulose without significant changing cellulose allomorph and crystallinity [17, 18]. Similar phenomena were described by many researchers, who demonstrated that irradiation pretreatment (e.g., *γ*-ray, plasma, electron beam, microwave) would become a universal approach to accelerate biomass degradation [19–21]. The mechanism was revealed that irradiation caused oxidative cleavage of glucan chain involved ROS. It is deduced that a synergistic effect may be existed between irradiation oxidative cleavage and enzyme hydrolysis for crystalline cellulose degradation. However, the oxidized microstructure of irradiated cellulose is unclear due to the low selectivity and complexity of physical oxidation. To the best of our knowledge, the synergistic effect on cellulose degradation between non-enzyme oxidative cleavage and hydrolytic cellulases has not been available so far.

To meet this challenge, first of all, it briefly describes the that high hydrolysis efficiency of crystalline cellulose is obtained after it is treated by IOPR process. To test the feasibility of IOPR in real biorefinery, several types of cellulose substrates, including “model” cellulose (e.g., Avicel PH-101) and real biomass cellulose (e.g., *Eucalyptus* hardwood and pine softwood), are investigated in this work. Then, using XRD, EPR, FTIR, XPS, GPC, MALDI-TOF, and HPLC techniques, it is comprehensively elucidated the fact that crystalline celluloses treated with IOPR show remarkable high degradation efficiency by cellulases, changes in nanoscale structural features of crystalline cellulose are systematically verified, including cellulose crystallinity index (CCI), accessibility, surface area, degree of polymerization (DP), number of reducing ends, functional group features during degradation, and oligosaccharide products. Furthermore, through TEM images, a digestion mechanism of crystalline cellulose by free hydrolytic cellulases is illustrated through IOPR process. Finally, the potential feasibility of IOPR process in an industrial setting is preliminarily discussed.

## Methods

### Materials

Avicel microcrystalline cellulose (PH-101, cellulose I), cellobiose (≥ 98%), ethylenediamine (≥ 99%), sodium azide, direct orange 15, direct blue 1, *Tr*Cel7A (0.13 U/mg powder, expressed in corn plant from *Hypocrea jecorina*), and Celluclast 1.5 L (145 FPU/mL, 369.4 mg protein/mL, Novozyme Corp.) were all purchased from Sigma Aldrich (St. Louis, MO, USA). *Tr*Cel7A and Celluclast 1.5 L were directly used from company without any purification. Additional file 1: Figure S1 shows the SDS-Page analyses of these enzymes. β-glucosidase (20.5 CBU/mg powder, 6.8 mg protein/g powder) was purchased from Jiangsu Ruiyang Biotechnology Co. Ltd. (Wuxi City, Jiangsu, China). Prior to experiments, the protein content in enzymes were verified by Bradford assay and BSA was used as standard.

Methanol and acetonitrile (HPLC grade) were purchased from Fisher Chemical Co. (Beijing, China). Ethanol, sodium hydroxide, glacial acetic acid, phosphoric acid, and sulfuric acid were of analytic grade and purchased from Beijing Chemical Factory (Beijing, China). Other reagents were purchased from local chemical company, Beijing, China.

### Cellulose substrates preparation

#### Cellulose substrate IC-I

Avicel PH-101 (~ 15 g) in a 20-mL glass tube was exposed to a gamma resource (^60^Co) with the level of 600 kGy at room temperature according to the procedures detailed in our previous work [22]. The irradiated Avicel (5 g) was suspended with 50 mL deionized water and stirred at room temperature for 1 h, and the mixture was filtered with 0.45-μm membrane. Then, the solid residue was washed with 50 mL deionized water for 3 times and lyophilized to obtain the irradiated cellulose I (defined as IC-I). The soluble fraction in the liquor was lyophilized and subjected to MALDI-TOF–MS and HPLC for sugars analyses. The untreated Avicel PH-101 substrate was defined as C-I in this work.

#### Cellulose substrate IpRC-I

The irradiated Avicel PH-101 without washing was used for post-reduction treatment with sodium borohydride (NaBH_4_) in alkali solution to prepare the substrate IpRC-I. The detailed procedures of post-reduction treatment were modified according to the method reported by Beeson et al. [23]. In brief, 0.6-g irradiated Avicel was mixed with 5 mL fresh preparation of 20 mg/mL NaBH_4_ in 1.0 M ammonium hydroxide. The reaction was preceded at room temperature for 2 h with occasional stirring. Then, 1 mL of glacial acetic acid was added to remove excessive NaBH_4_. 10 mL of methanol:acetic acid (9:1, vol.%) mixtures was added to neutralize the alkali and it stopped the reduction reaction. Finally, the mixture was centrifuged at 8000 rpm for 5 min; the solid residue was washed with 50 mL methanol in triplicate up to pH neutral, and air-dried over 12 h to achieve cellulose powder, which was defined as cellulose substrate IpRC-I. The methanol in the supernatant was removed with N_2_ at 40 °C, and then lyophilized for sugars analyses by HPLC.

#### Cellulose substrate C-II

Avicel PH-101 (10 g) was dipped into 100 mL 25% NaOH (w/v) solution at 4 °C for 1 h. The slurry was then centrifuged at 5000 rpm for 30 min, and washed with 150 mL of cool deionized water for 6 times up to neutral pH [24]. The washed solid residue was lyophilized and defined as cellulose substrate C-II for subsequent experiments.

#### Cellulose substrate C-III

Avicel PH-101 (5 g) was soaked in 100 mL of ethylenediamine at room temperature for 24 h. The slurry was then centrifuged at 5000 rpm for 30 min, and washed with anhydrous methanol (6 × 150 mL) until neutral pH [25]. The washed solid residue was air-dried and defined as cellulose substrate C-III for subsequent experiments.

#### Cellulose substrates C-E and C-P

Two crystalline cellulosic substrates from real biomass, *Eucalyptus* hardwood and pine softwood, were prepared and defined as C-E (85% glucan content) and C-P (78% glucan content), respectively. The detail procedures of cellulose substrates C-E and C-P preparation were described in our previous work [17]. In brief, the irradiated wood biomass (88 g, 800 kGy) was added into 350 mL of aqueous *γ*-valerolactone solution (40:60 GVL/H_2_O, vol%); the mixture was heated to 170 °C and kept for 1 h. After cooling, the slurry was filtered to obtain the solid residue, which was successively washed with aqueous *γ*-valerolactone (3 × 300 mL) and deionized water (3 × 500 mL). The crystalline allomorph of cellulose substrates C-E and C-P was confirmed by XRD, and the glucan contents of C-E and C-P were analyzed by HPLC.

### Enzymatic hydrolysis of cellulose substrates

Enzymatic hydrolysis of cellulose substrates by sole *Tr*Cel7A was performed with 0.5% (w/v) glucan in 50 mM acetate buffer (pH 5.0, 25 mg/L of sodium azide) at enzyme loading of 12 mg protein/g glucan. The enzyme hydrolysis time was fixed at 0.5, 1, 2, 3, 5, 7, and 9 h. The hydrolysis of cellulose by free hydrolytic cellulases (90% Celluclast 1.5 L and 10% β-glucosidase) was carried out at 1% (w/v) glucan loading with different enzyme loadings (2, 5, 10, 20, and 50 mg protein/g glucan) for 6, 12, 24, 36, and 48 h. The content of cellulose substrate loading for enzymatic hydrolysis was monitored on the basis of the glucan content in substrate. Other enzyme hydrolysis conditions were as follows: reaction volume 1 mL, temperature 50 °C, and agitation 60 rpm in an end-over-end rotary incubator. The enzyme hydrolysis reaction was immediately stopped by putting reactor in the boiling water bath for 5 min. The hydrolysates were subjected to HPLC analysis. All experiments were repeated in triplicate and the final experimental data were expressed as mean ± StDev in this work.

### Enzyme adsorption assays of cellulose substrates

Cellulases (*Tr*Cel7A and Celluclast 1.5 L) adsorption assays were performed according to the procedures reported by Chundawat et al. [24]. In brief, 10 mg of cellulose substrate was suspended in 50 mM acetate buffer (pH 5.0) in a 2-mL centrifuge tube with the total volume of 1 mL. Then, a certain amount of cellulase (1–150 mg protein/g cellulose) was added to the tube for incubation at 4 °C, and stirred with 60 rpm in end-over-end rotation for 2 h. Through centrifugation at 4 °C and 10,000×*g* for 5 min, the supernatant was used for protein content measurement by Bradford assay using BSA as standard [25]. Langmuir equation (Eq. 1) was used to calculate enzyme adsorption content using PASW Statistics software (Version 18.0, IBM SPSS, Inc., USA).

1 $$ [B]\, = \,\frac{{B_{\text {max} } \, \times \,[F]}}{{K_{d} \, + \,[F]}}, $$

where [*B*] is the amount of bound cellulase (mg protein/g cellulose), calculated as (loaded protein-total protein in supernatant)/loaded cellulose. *B*_max_ is the maximum cellulase binding capacities for cellulosic substrate. [*F*] is the free enzyme concentration in supernatant (mg/mL). *K*_*d*_ is the apparent dissociation constant.

### Accessibility of cellulose substrates by Simons’ stain

The accessibility of cellulases to cellulose substrates (C-I, IC-I, IpRC-I) was evaluated by Simons’ stain [26]. Since the molecular size of orange dye’s (5–36 nm) is almost the same as that of typical cellulase, the adsorption of orange dye during Simons’ stain is normally used to determine the accessible surface area of cellulose substrate [27].

### Electron paramagnetic resonance (EPR) monitoring activation oxygen radicals

EPR experiments were performed on a JES FA-200cw-EPR spectrometer (JEOL, Japan) at room temperature to monitor the free radicals generating from irradiation oxidation towards cellulose. The detailed procedures were described by Liu et al. [28]. The EPR conditions were as follows: microwave frequency (x-band) 9.06 GHz, microwave power 10 mW, center field 324 mT, sweep width 50 mT, modulation amplitude 0.35 mT, modulation frequency 100 kHz, sweep time 60 s, and temperature 37 °C. The EPR spectra were normalized based on the mass of the sample.

### Attenuated total reflectance Fourier transform infrared spectroscopy (ATR-FTIR)

The variances of functional groups towards cellulose substrates C-I, IC-I, and IpRC-I were analyzed by FT-IR (TENSOR 27, Bruker, USA) equipped with a temperature-controlled attenuated total reflectance (ATR) device with a ZnSe crystal (Pike Technology) [29]. The spectra were recorded from 600 to 4000/cm at a resolution of 2/cm.

### X-ray diffraction (XRD)

The crystallinity of cellulose substrates was performed on a D8 ADVANCE XRD (Bruker, Germany) using Cu K*α* radiation at a voltage of 40 kV and a current of 40 mA. Scans were obtained from 2*è* = 5 to 45, 30 min per sample. The cellulose crystallinity index (CCl) was commonly estimated using two methods: XRD peak height and XRD peak deconvolution [24, 30].

In case of XRD peak height method, cellulose crystallinity index (abbrev. CCI_H_) was calculated using Eq. (2) from the intensity of the 002 peak (*I*_002_) height and the minimum (*I*_AM_) height between the 002 and 101 peaks.

2 $$ {\text{CC1}}_{H} \, = \,\frac{{I_{002} \, - \,I_{AM} }}{{I_{002} }}\, \times \,100, $$

where *I*_002_ is the peak height at 2*θ* = ~ 22.5°; *I*_AM_ is the peak height at 2*θ* = ~ 18.6°.

For the XRD peak deconvolution method, cellulose crystallinity index (abbrev. CCI_D_) was estimated by the percent ratio of the crystalline peaks (A_C_) to the total area (A_T_) of all deconvoluted peaks, which is expressed as follows:

3 $$ {\text{CC1}}_{D} \, = \,\frac{{A_{C} }}{{A_{T} }}\, \times \,100. $$

Peak deconvolutions were performed using PeakFit (Version 4.12, Systat Software Inc, San Jose, CA). XRD experimental data were fitted using Gaussian–Lorentzian analysis to sure *F* value > 30,000 and *R*^2^ > 0.998. For crystalline cellulose I, five crystalline peaks (at 2*θ* = 14.8°, 16.5°, 20.5°, 22.5°, and 34.5°) and one amorphous peak (2*θ* = 21.5°) were deconvoluted and fitted to obtain the XRD original spectra.

### Gel permeation chromatography (GPC)

Cellulose substrates C-I, IC-I, and IpRC-I were derivatized with anhydrous pyridine and phenyl isocyanate prior to GPC analyses for the molecular weight distribution [27]. A weighted cellulose substrate was dissolved in tetrahydrofuran (abbrev. THF) with the final concentration of 5 g/L. GPC analyses were performed on 1515GPC instrument (Waters, USA) equipped with refractive index (abbrev. RI) detector. The conditions were as follows: THF was used as eluent at the rate of 1.0 mL/min; injection volume was 50.0 ìL. Column temperature was 35 °C. The detected molecular weight ranges were from 500 to 4 × 10^6^ Da. The average molecular weight was calculated by the universal polystyrene calibration curve [27]. Degree of polymerization (DP) was calculated using these molecular weights divided by 519, the molecular weight of cellulose tricarbanilate monomer [31].

### Transmission electron microscopy (TEM)

To elucidate the effect of IPOR process on the enzyme hydrolysis mechanism, the morphologies of cellulose substrates C-I, IC-I, and IpRC-I after enzymatic digestion were imaged by 120 kV TEM (HT7700, Hitachi, Japan) with a 4 mega-pixel Gatan UltraScan 1000 camera (Gatan, Pleasanton, CA). For TEM images, the samples were dropwise cast directly on carbon-coated copper grids, and negatively stained with 2 wt% aqueous phosphotungstic acid (pH 6.2) [32].

### X-ray photoelectron spectroscopy (XPS)

Elemental distribution and chemical state on the surface (5–10 nm) of cellulose substrates C-I, IC-I, and IpRC-I were analyzed by ESCALAB 250 XPS (Thermo Fisher Scientific, USA). The data were acquired using twin anode Al Kalph (300 W), a pass energy of 100 eV for survey; 30 eV for high-resolution scans. The analyzed area was 500 ìm × 500 ìm. The carbon element signals were deconvoluted (within 0.2 eV) into C1 (284.8 eV), C2 (286.5 eV), C3 (287.9 eV), and C4 (288.8 eV) signals using XPS PEAK (Version 4.1) [33].

### Matrix-assisted laser desorption/ionization-time of flight–mass spectrometry (MALDI-TOF–MS)

MALDI-TOF–MS was performed to detect the oligosaccharides concentration on an ABI 4700 Voyager DE PRO (USA), using 2,5-dihydroxybenzoic acid (DHB) as matrix. 8 μL of DHB solution (10 mg/mL) was mixed with 2 μL of oligosaccharides solution (10 mg/mL). 0.5 μL of mixture solution was deposed on a MTP 384 target plate ground. The spotted samples were then dried in a vacuum desiccator. The spectra were obtained using the reflection mode with an acceleration voltage of 25 kV, a reflector voltage of 26, and pulsed ion extraction of 40 ns in the positive ion mode. The acquisition range was from m/z 500 to 4000 [12].

### Trifluoroacetic acid hydrolysis for oligosaccharides determination

To quantification of soluble oligosaccharides derived from IOPR, the samples were completely hydrolyzed by trifluoroacetic acid (TFA) prior to HPLC analysis [23]. Specifically, the lyophilized oligosaccharides (10 mg) were dissolved in 2 mL aqueous TFA (2.0 M) and hydrolyzed in a preheated oil bath at 121 °C for 1 h. After reaction, the samples were immediately cooled with ice water, and centrifuged at 8000×*g* for 5 min. The supernatant was dried under a stream of nitrogen at 40 °C. The dried sample was duplicate washed with 5 mL isopropanol and centrifuged at 10,000×*g* for 5 min, then the precipitate was dried under a stream of nitrogen. The final samples were dissolved in water for monomeric sugars detection by HPLC.

### High-performance liquid chromatograph (HPLC)

The quantification of cellobiose and glucose was performed on a Series 1500 HPLC (Alltech,USA) with a prevail carbohydrate ES 5 μ column (GRACE) and an evaporative light-scattering detector (ELSD). The mixture solution of acetonitrile:H_2_O = 75:25 (v/v) was used as the mobile phase at the flow rate of 1 mL/min. The column temperature was 30 °C.

The xylose, glucose, and sorbitol were also analyzed by HPLC with refractive index detector (RI, Schambeck SFD GmbH,Germany). The separation was performed in an Aminex HPX-87H column (BIO-RAD) at 60 °C. The mobile phase was 5 mM H_2_SO_4_ at a flow rate of 0.6 mL/min [34]. All samples were filtered with 0.22-μm membrane prior to HPLC analysis.

## Results and discussions

### Irradiation and IOPR process boost the enzymatic degradation of recalcitrant crystalline cellulose

**Fig. 1:**
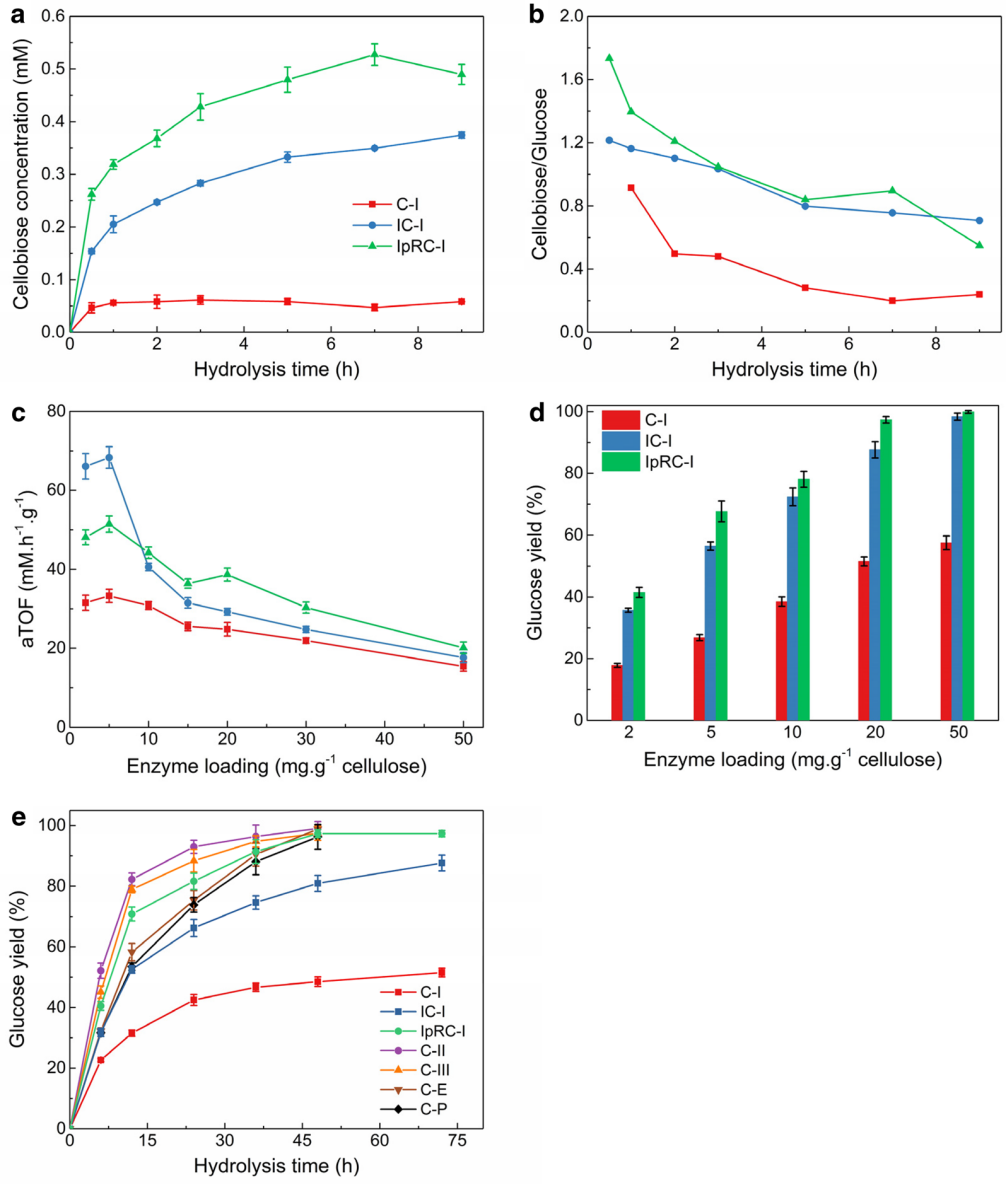
The enzyme hydrolytic performance of different cellulose substrates. **a** Cellobiose accumulation. **b** cellobiose/glucose ratio from cellulose by *Tr*Cel7A alone (12 mg enzyme*/*g glucan). **c** TOF of cellulase at different enzyme loadings. aTOF is apparent turnover frequency, which is defined as the mole number of glucose production per hour per gram enzyme at initial hydrolytic stage. **d** Effect of cellulases loadings on glucose release for 72-h hydrolysis. **e** The kinetics curve of glucose release during enzymatic hydrolysis under the enzyme loading of 20 mg protein/g-cellulose. Other conditions are as follows: cellulose substrate concentration 1%, 50 mM acetate acid buffer (pH 5.0), cellulases complexes of 90% Celluclast 1.5 L and 10% β-glucosidase

Avicel cellulose was subjected to gamma-ray irradiation followed by reduction with sodium borohydride to enhance enzymatic degradability. The untreated (C-I), irradiated (IC-I), and irradiated and borohydride-treated (IpRC-I) cellulose materials (0.5% w/v) were incubated at pH 5.0 and 50 °C, with either *T. reesei* Cel7A (TrCel7A) alone, or a cellulase enzyme mixture (90% Celluclast 1.5 L, 10% beta-glucosidase), and the amount of glucose and cellobiose released over time was monitored by HPLC. The results are shown in Fig. 1.

In comparison of the control (C-I), cellulose substrates IC-I and IpRC-I are easily more prone to degradation by cellulases, which are dramatically releasing higher concentration of cellobiose (Fig. 1a) and ratio of cellobiose/glucose (Fig. 1b). The ratio of cellobiose/glucose for IC-I and IpRC-I increases with the rate of cellulobiose during the hydrolysis. It indicates that IC-I and IpRC-I are more prone to degradation by CBH I to release cellobiose. In other words, cellobiohydrolase shows the higher hydrolytic velocity on IC-I and IpRC-I [35].

Furthermore, the apparent turnover frequency (TOF) of free hydrolytic cellulases (Celluclast 1.5 L and β-glucosidase) was also investigated in our work. An interesting observation in Fig. 1c shows that TOF for IC-I hydrolysis is higher than these for IpRC-I and C-I at low enzyme loadings (2–5 mg protein/g glucan). It is perhaps the hypothesis that irradiation oxidative cleavage of glycosidic bonds in crystalline cellulose opens up the inaccessible oligosaccharides for cellulases [3]. It is easily deduced that high glucose concentration from the irradiated biomass and/or “model” cellulose can be probably obtained at relatively low enzyme dosage. This phenomenon was confirmed in our previous work, wherein 76% of cellulose conversion to glucose was achieved for high crystalline cellulose derived from irradiated lignocellulose at relatively low enzyme loadings of ~ 7 mg protein/g cellulose for 52-h hydrolysis [18].

Alternatively, we analyzed the effects of cellulase loadings ranging from 2 to 50 mg protein/g cellulose on glucose release of C-I, IC-I, and IpRC-I biodegradation (Fig. 1d). The effect of enzyme loadings on the initial rate of glucose release from C-I, IC-I, and IpRC-I was evaluated during enzymatic hydrolysis, and the results are depicted in Additional file 1: Figure S2. As expected, the observed results of Fig. 1d reveal that the highest glucose yields released from IpRC-I cellulose are achieved, following with IC-I. However, C-I releases the lowest glucose yields under the same conditions. Here is a specific example, a total enzyme loading of 20 mg per g cellulose is used to ensure that about 97% of IpRC-I and 86% of IC-I substrates can be converted into glucose for 48-h and 72-h cellulase hydrolysis, respectively, while only 43% of C-I is converted into glucose.

In addition, the kinetics curves of cellulase hydrolysis on different cellulose substrates were investigated, including crystalline cellulose II, III, cellulose derived from hardwood *Eucalyptus* treated with irradiation (defined as C-E), and softwood pine treated with irradiation (defined as C-P) under the same conditions. As shown in Fig. 1e, irradiation and/or IOPR treatments are able to enhance enzyme hydrolysis efficiency regardless of real lignocellulose biomass and/or “model” cellulose. It was reported that cellulose III was hydrolyzed by fungal cellulases from *T. reesei* at rates up to 5-fold higher than native cellulose I [24]. It is the common conception that crystalline cellulose I was considered as much harder recalcitrance to enzymatic hydrolysis than other allomorph forms (II, III, and PASC) due to highly ordered glucan chains and each chain stabilized by intra-/inter-molecular hydrogen bonds in the crystal [24]. Excitedly, all tested cellulose substrates after IOPR treatment in our work are completely digested for 48-h enzyme hydrolysis. It has been demonstrated that IOPR process facilitates enzymatic degradation of recalcitrant crystalline cellulose, which is a potential competitive alternative to current biomass pretreatment for the real biorefinery [17, 18, 22].

### Structural characteristics of crystalline cellulose treated by irradiation and IOPR process

**Fig. 2:**
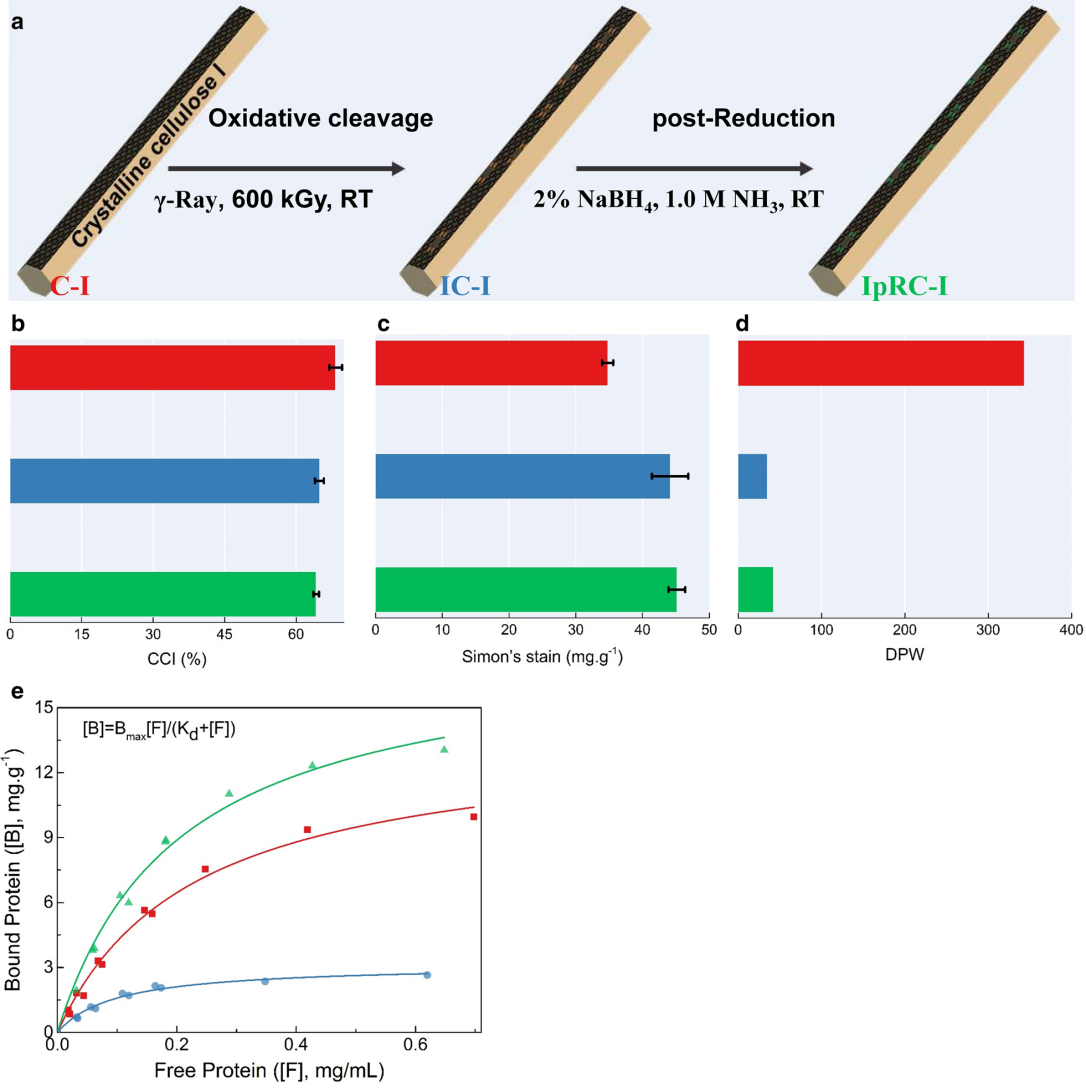
Schematic illustration and physico-chemical properties of prepared cellulosic substrates. **a** Procedures for preparation of IC-I and IpRC-I. **b** Cellulose crystallinity index (CCI). **c** Accessibility. **d** Degree of polymerization (DP), and **e** Enzyme adsorption of *Tr*Cel7A and Celluclast 1.5 L

To elucidate the fact that crystalline celluloses treated with IOPR show remarkable high degradation efficiency by cellulases, changes in structural features of crystalline cellulose, such as cellulose crystallinity index (CCI), accessibility, surface area, degree of polymerization (DP), number of reducing ends, functional group features during degradation, and oligosaccharide products, are systematically verified through many model techniques, including XRD, EPR, ATR- FTIR, GPC, XPS, MAIDI-TOF–MS, and HPLC. The flow scheme of IOPR process was shown in Fig. 2a.

The observed data in Fig. 2b show that IOPR treatment does not alter its allomorph form of crystalline cellulose I. However, after stepwise irradiation oxidation and NaBH_4_ reduction treatment, crystalline cellulose I results in a 4.98 and 6.0% decrease of CCl, respectively (Fig. 2b). It was pointed out that irradiation and IOPR had slight influence on allomorph and crystallinity of cellulose [21]. This phenomenon is confirmed by X-ray diffraction (XRD) analyses (Additional file 1: Figure S3) and is good consistent with these reported in the literatures [21, 36]. The characteristic peaks of XRD patterns for C-I, IC-I, and IpRC-I were observed at 14.8°, 16.5°, 20.5°, 22.5°, and 34.5°, which are corresponding to crystalline peak 101, 10ī, 021, 002, and 040 of cellulose allomorph I, respectively (Additional file 1: Figure S3**)**.

The enzyme accessibility of cellulosic substrates, C-I, IC-I, and IpRC-I, was evaluated by Simon’s stain method [27]. The observed data in Fig. 2c show that IC-I and IpRC-I achieve a 26.7 and 29.6% of enhancement in accessibility, respectively, in comparison with C-I. It may be attributed to the fact that irradiation induces partial degradation of cellulose to soluble oligosaccharides (Additional file 1: Figure S4) according to HPLC analyses. It is speculated that irradiation can cleave the backbone-chain structure of crystalline cellulose, which was confirmed by GPC analyses. The observed data in Fig. 2d show that irradiation causes dramatic decrease in the cellulose DPw from 343 to 35 ~ 42. It suggests that IC-I and IpRC-I have 9.5- and 8-fold times of total number of chain ends over C-I substrate (Additional file 1: Figure S5). The reasonable explanation may be the fact that irradiation generates activation oxygen radicals resulting in 1,4-glucan chain cleavage of cellulose [37]. The derived radical oxygen species (ROS) was further confirmed from EPR analyses (Additional file 1: Figure S6).

Generally, the increased accessibility and new chain ends of IC-I and IpRC-I provide more opportunity for enzyme binding. However, although the molecular size of orange dye’s (5–36 nm) is approximately similar to the typical cellulases, the data of cellulose accessibility by Simon’s stain method are not consistent with the data of enzyme adsorption on cellulose surface. For instance, the data in Fig. 2e and Table 1 confirm that the cellulase adsorption on IC-I substrate is much lower than C-I. After NaBH_4_ reduction process, the cellulase adsorption on IpRC-I is higher than C-I. It is speculated that there exists some substrate-disrupting factors making IC-I not accessible to enzyme molecular [12]. These substrate-disrupting factors are demonstrated to be gluconic acid compounds according to the report by Arfi et al. [13]. After post-reduction treatment, these “substrate-disrupting” factors are transferred into “substrate-affinity” factors, which are more accessible to glycosidic hydrolases.

**Table 1:** Adsorption ability of *Tr*Cel7A and Celluclast 1.5 L for three cellulosic substrates

	Adsorption parameters for *Tr*Cel 7A			*B*_max_ for Celluclast 1.5 L (mg/g)
	*B*_max_ (mg/g)	K_d_	Adj. *R*^2^	
C-I	13.79	0.23	0.991	21.02
IC-I	3.14	0.10	0.982	4.91
IpRC-I	17.88	0.20	0.990	25.61

To further explain “substrate-affinity” factors, XPS and FT-IR experiments were conducted. The data in Additional file 1: Figures S7 and S8 show that weak acid groups (e.g., aldehydes, ketones, lactones) of cellulose are formed after irradiation treatment. After post-reduction, these acid groups are reduced by sodium borohydride and transferred into hydrogen group. These new reduced end existing in cellulose structure may be easily accessible to cellulases. Recently, a similar phenomenon was reported by Hu et al. [37] that the addition of LPMO (AA9) to a commercial cellulase mixture would decrease Cel7A (a processive exoglucanase) adsorption during hydrolysis of crystalline cellulose I. Nakamura and co-workers [35] pointed out that the hydrolytic velocity of CBH I on the crystalline cellulose surface was related to a high dissociation rate constant from the substrate.

Based on the structure characteristics of cellulose substrates, C-I, IC-I, and IpRC-I, it was observed that β-1,4 glycosidic bonds of cellulose backbone chain were scissored by ROS derived from irradiation oxidation. XPS analysis showed that a range of new oxidized end products is formed with ketone group (–C=O–) when cellulose was treated by irradiation oxidation (e.g., IC-I cellulose), while FT-IR data reveal that a range of new chain reductive end products is generated and ketone functional group is disappeared after post-reduction treatment (e.g., IpRC-I cellulose). These new reductive end products may be acting as “substrate-affinity” factors much easier accessible to cellulases. Therefore, a positive effect on hydrolytic efficiency (seen in Fig. 1) is achieved by the oxidized and reduced new chain ends, which damage the normal chair conformation of the sugar ring [12].

### Analysis of the new chain end products of crystalline cellulose treated with irradiation and IOPR process

**Fig. 3:**
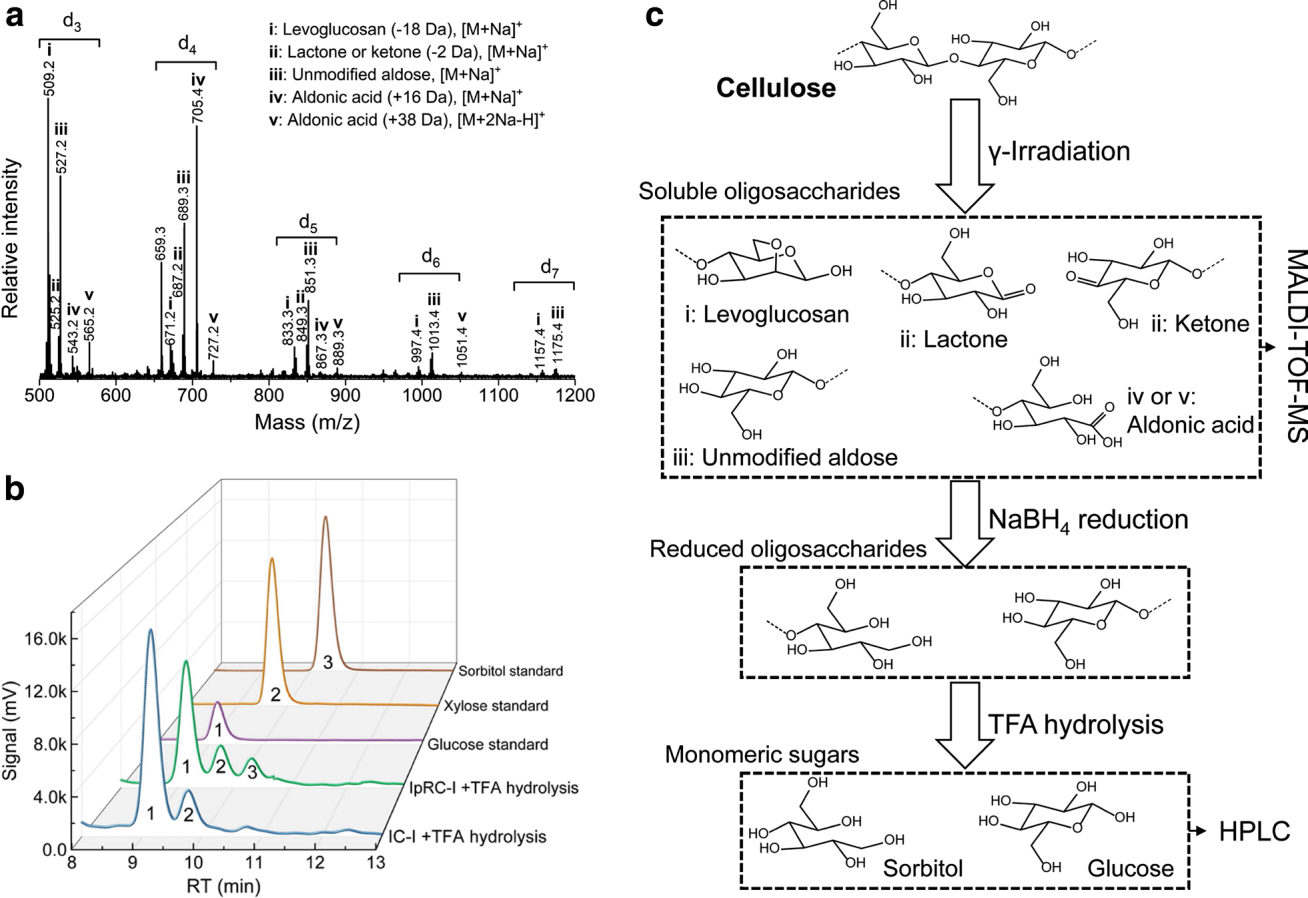
Products identification of crystalline cellulose treated by irradiation and IOPR process. **a** MALDI-TOT-MS spectra of soluble oligosaccharides from irradiated cellulose, where d_x_ indicate the DP of the oligosaccharides, i, ii, iii etc. indicate the product species. **b** HPLC chromatogram of soluble oligosaccharides produced by irradiation and IOPR process followed by TFA hydrolysis. **c** Schematic showing the experimental design for identification of irradiation caused chain cleavage

To clearly resolve the new glucan chain ends of cellulose structure, the soluble oligosaccharides from washing steps in irradiation and IOPR process were analyzed by MALDI-TOF–MS and HPLC [23]. The data of MALDI-TOF–MS in Fig. 3a reveal a series of molecular ions corresponding to modified oligosaccharides with degree of polymerization (DP) from 3 to 7. Higher DP ≥ 8 of cellulose-oligosaccharides are not detected in this instrument. It suggests that irradiation oxidation scission as indicative of an “endo”-type of activity cleaving glycosidic bond [12, 38]. Notwithstanding longer oligosaccharides are difficult to detect due to their low solubility, however, we cannot hitherto confirm whether irradiation oxidation acts randomly on crystalline surfaces or show a strong preference for cleaving glycosidic bond. Five main species were observed for each oligosaccharide: the respective levoglucosan ([M + Na] +, − 18 Da) or monosodiated or disodiated aldonic acid ([M + Na] +, + 16 Da; [M + 2Na–H] +, + 38 Da) or lactone ([M + Na] +, − 2 Da) or unmodified oligosaccharides ([M + Na] +, + 0 Da). The proposed structure models of oligosaccharides were shown in Fig. 3c. Expectedly, the oxidized products (e.g., lactones, aldonic acids) generated from irradiation oxidation were detected as “substrate-disrupting” factors. The patten of oxidized oligomers (DP from 3 to 7) generated by irradiation oxidation is similar to the pattern as metal oxygenases (e.g., LPMOs) that oxidatively break down recalcitrant crystalline cellulose to oligosaccharides [14, 23, 39]. Interestingly, some unmodified oligosaccharides and levoglucosan were observed in the products of irradiation oxidative cellulose; these unmodified oligosaccharides were derived from glycosidic bonds hydrolysis and the intramolecular dehydration of glucose. Unlike LPMOs oxidation, irradiation-induced ROS caused oxidative cleavage and/or hydrolysis of glycosidic bonds of cellulose with low selectivity. The data in Fig. 3b show that sorbitol is detected as “substrate-affinity” factor after IOPR process. It suggested that sorbitol structure existed at the glucan chain ends of cellulose after IOPR process due to the reductive hydrolysis of lactones [23].

From these above observations, two important features stand out. First, a cleavage of glycosidic bond in crystalline cellulose after IOPR treatment is achieved by conjugating with an ROS oxidation step. Second, new chain end products of sorbitol structure released from IOPR are dominated by even-numbered oligosaccharides with the DP ≤ 7. The schematic overview of irradiation and IOPR related to cleavage of glycosidic bonds of crystalline cellulose association with oxidized and reduced new chain ends are shown in Fig. 3c. These nanoscale architecture modifications of cellulose reveal a synergy linkage of IOPR and cellulase hydrolysis, wherein the degradation mechanism of cellulose shows noticeable difference from the traditional glycoside hydrolysis [40].

### Fascinating digestion mechanism of crystalline cellulose by free hydrolytic cellulases after irradiation and IOPR process

**Fig. 4:**
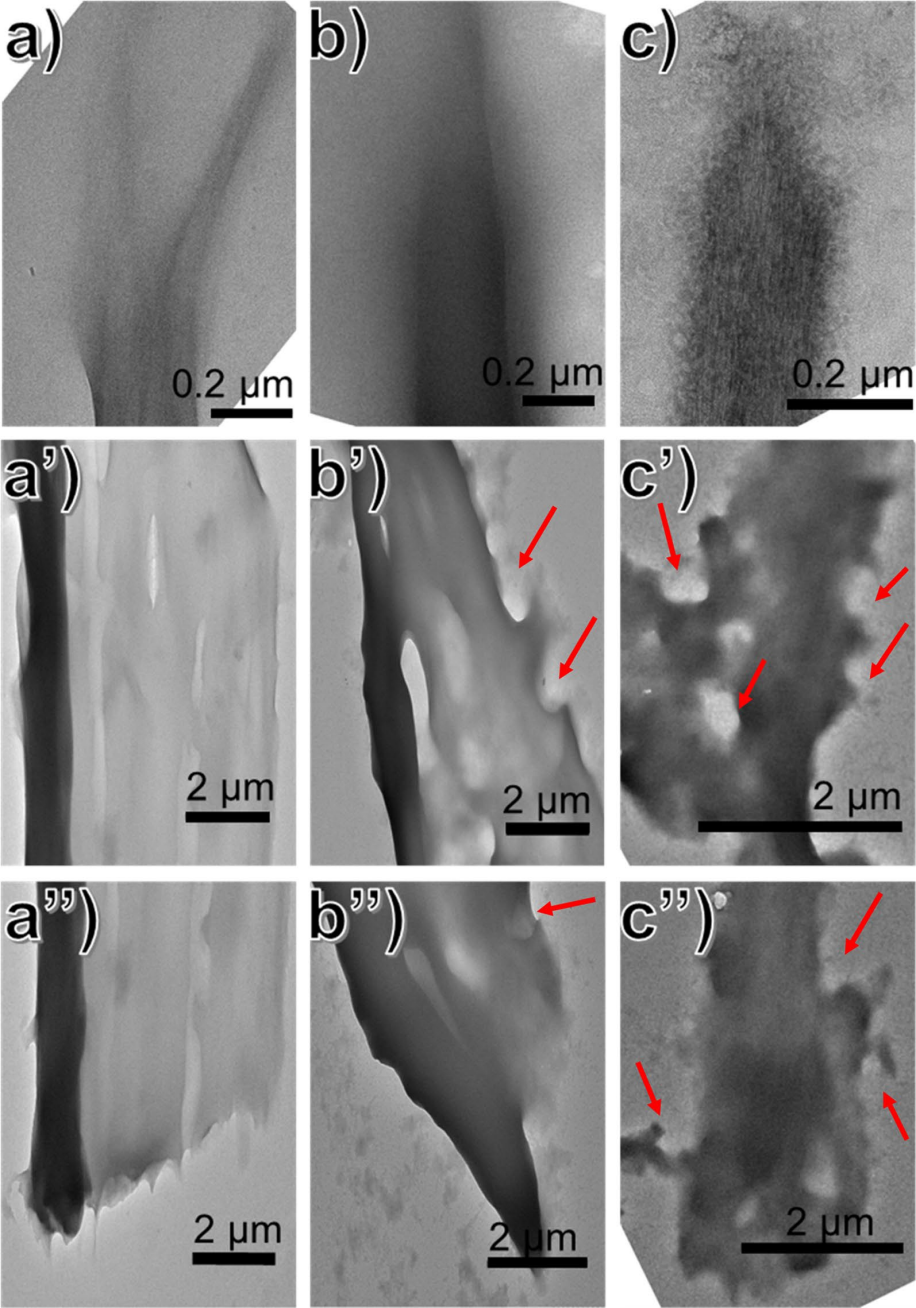
TEM micrographs of partially digested C-I, IC-I, and IpRC-I cellulose particles. That cellulose was digested for 24 h by cellulases cocktail. Avicel C-I (**a**, **a’**, **a’’**) digested to 42.5% conversion. Cellulose IC-I (**b**, **b’**, **b’’**) digested to 66.2% conversion. Cellulose IpRC-I (**c**, **c’**, **c’’**) digested to 81.6% conversion

It is inferred that the overall performance of cellulases acting on IC-I and IpRC-I surface is different from their actions on C-I substrate. The differences in the digestion mechanism by free hydrolytic cellulases (Celluclast 1.5 L and β-glucosidase) were imaged by TEM [32, 35, 39, 41]. The results are illustrated in Fig. 4 and Additional file 1: Figure S9.

TEM images in Fig. 4 confirm that there exists a fascinating digestion mechanism of “peel-off” and “cavity-form” combination for cellulases acting on crystalline cellulose after cellulose substrates are treated with IOPR process. In case of irradiated cellulose (IC-I), the reducing end of the particle is finely tapered to a narrow point, and the opposite end displays a blunt edge, exhibiting a slight angle from the long axis (Fig. 4B, B’’), and the middle surface shows ablation (Fig. 4B’). As for the native cellulose substrate (C-I), the similar phenomenon is observed for both ends of cellulose hydrolyzed by cellulases (Fig. 4A, A’’), but the middle keeps almost undamaged (Fig. 4A’; Additional file 1: Figure S9). This results in reducing end-oriented digestion mechanism on the surface as Ce17A from *T. reesei* [12]; it is the typical “peel off” mechanism that CBH I (e.g., *Tr*Cel7A) degrades the cellulose from the reducing end, narrowing of the crystal end, whereas CBH II (e.g., *Tr*Cel6A) hydrolyzes the cellulose chain from the nonreducing end less processively than CBH I, sharpening the crystal tip [39, 41].

Regarding NaBH_4_ post-reduction cellulose (IpRC-I), TEM images show that the reducing end of the particle displays narrowed, irregular, but not finely tapered morphology (Fig. 4c), and cavities-formation is observed in the middle and the opposite end (Fig. 4c’, c’’). It can be attributed to the fact that the new end products in glucan chain generated from IOPR are much accessible to CBHs, which excavate extensive cavities into the surface of the cellulose bundles. This fascinating digestion paradigm is similar to the cavity-forming mechanism caused by cellulosomes (e.g., Ce17A from *Caldicellulosiruptor bescii*) [32].

In combination with MALDI-TOF–MS and TEM, a reasonable conclusion can be drawn that there is an unexpected connection between cellulose chain structural modification, especially chain end products, and enzymatic degradation of crystalline cellulose, rather than only focusing on the traditional concerns about DP, accessibility, crystallinity and allomorphs forms of cellulose. This encouraging result opens a new avenue toward the implementation of pretreatment and the design of enzyme cocktails in lignocellulosic biorefinery.

## Conclusions

A novel non-enzymatic oxidative cleavage strategy of IOPR process is proposed to act on crystalline cellulose I to boost downstream degradation by cellulases from *Trichoderma reesei.* Regardless of the “model” cellulose substrates (e.g., Avicel PH-101) or real biomass cellulose (e.g., *Eucalyptus* and Pine), the treated crystalline celluloses are easily bio-converted into fermentable sugars. 81.6% of conversion efficiency was achieved for 24-h cellulase hydrolysis of crystalline cellulose. Assuming elongation of saccharification time to 48 h, 97% of conversion efficiency has been reached. Combining consideration of the low milling energy consumption to the crackdown of feedstock after irradiation pretreatment on biomass [18, 22, 42], a practical cost-efficient pretreatment protocol will be suggested for lignocellulosic biorefinery. Although the comprehensive assessment of IOPR process still needs to experimentally evaluate the potential feasibility in an industrial setting, the results suggest that the proposed approach is motivating Scientists inspiration and will become an economically competitive alternative to current biomass pretreatment.

## Additional file

**Additional file 1: Figure S1.** SDS-Page of *Tr*Cel 7A and Celluclast 1.5 L. **Figure S2.** Composition of irradiated Avicel. **Figure S3.** Effect of enzyme loadings on the initial rate of glucose release during enzymatic hydrolysis. **Figure S4.** XRD patterns of C-I, IC-I and IpRC-I cellulose substrates. **Figure S5.** GPC of C-I, IC-I and IpRC-I cellulose substrates. **Figure S6.** EPR of crystalline cellulose substrate before and after irradiation treatment. **Figure S7.** XPS of C-I, IC-I and IpRC-I cellulose substrates. **Figure S8.** ATR-FTIR of C-I, IC-I and IpRC-I cellulose substrates. **Figure S9.** Additional TEM micrographs of partially digested C-I, IC-I and IpRC-I cellulose particles.

## Abbreviations

ATR-FTIR: attenuated total reflectance Fourier transform infrared spectroscopy
CBH: cellobiohydrolase
EG: endoglucanase
EPR: electron paramagnetic resonance
GPC: gel permeation chromatography
HPLC: high-performance liquid chromatograph
LPMOs: lytic polysaccharide monooxygenases
MALDI-TOF–MS: matrix-assisted laser desorption/ionization-time of flight–mass spectrometry
ROS: reactive oxygen species
TEM: transmission electron microscopy
XPS: X-ray photoelectron spectroscopy
XRD: X-ray diffraction
