# Correction to: Physico-chemical oxidative cleavage strategy facilitates the degradation of recalcitrant crystalline cellulose by cellulases hydrolysis

**DOI:** 10.1186/s13068-018-1109-9

**Published:** 2018-04-11

**Authors:** Hua Zhou, Liuyang Wang, Yun Liu

**Affiliations:** Beijing Key Laboratory of Bioprocess, College of Life Science and Technology, Beijing University of Chemical Technology, Beijing, 100029 China

## Correction to: Biotechnol Biofuels (2018) 11:16 10.1186/s13068-018-1016-0

The original version of this article [1] unfortunately contained a mistake in the author’s group. The given name of “Hua Zhou”, was misspelled as “Huan Zhou”. The coauthor name is corrected with this erratum.
