# Supplementary material for: Physico-chemical oxidative cleavage strategy facilitates the degradation of recalcitrant crystalline cellulose by cellulases hydrolysis
Source: Biotechnol Biofuels. 2018 Jan 25;11:16. doi: 10.1186/s13068-018-1016-0 (PMC5784611; doi:10.1186/s13068-018-1016-0)
Supplement: Supplementary file 1 — Additional file 1: Figure S1. SDS-Page of TrCel 7A and Celluclast 1.5 L. Figure S2. Composition of irradiated Avicel. Figure S3. Effect of enzyme loadings on the initial rate of glucose release during enzymatic hydrolysis. Figure S4. XRD patterns of C-I, IC-I and IpRC-I cellulose substrates. Figure S5. GPC of C-I, IC-I and IpRC-I cellulose substrates. Figure S6. EPR of crystalline cellulose substrate before and after irradiation treatment. Figure S7. XPS of C-I, IC-I and IpRC-I cellulose substrates. Figure S8. ATR-FTIR of C-I, IC-I and IpRC-I cellulose substrates. Figure S9. Additional TEM micrographs of partially digested C-I, IC-I and IpRC-I cellulose particles. [file 13068_2018_1016_MOESM1_ESM.docx]

**Additional files**

**Physico-chemical oxidative cleavage strategy facilitates the degradation of recalcitrant crystalline cellulose by cellulases hydrolysis**

Hua Zhou, Liuyang Wang, Yun Liu^*^

Beijing Key Laboratory of Bioprocess, College of Life Science and Technology, Beijing University of Chemical Technology, Beijing 100029, China

Figs. S1 to S8


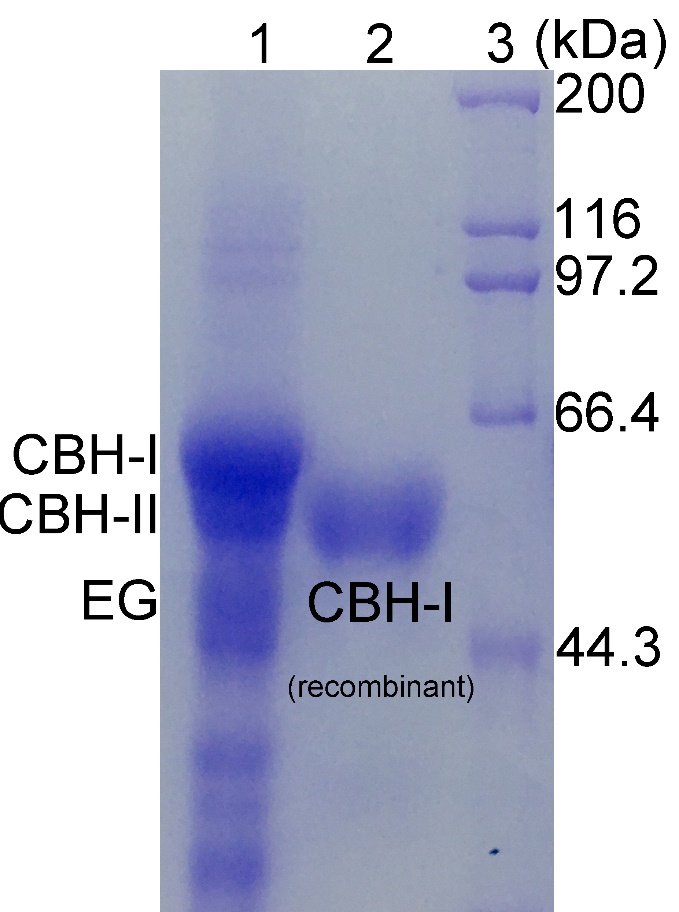


**Fig. S1** SDS-Page analysis of enzyme. Lane 1 was the Celluclast 1.5L from *T. reessei*; Lane 2 was the *TrCel*7A recombinant in corn plant; Lane 3 was the marker molecular weight.


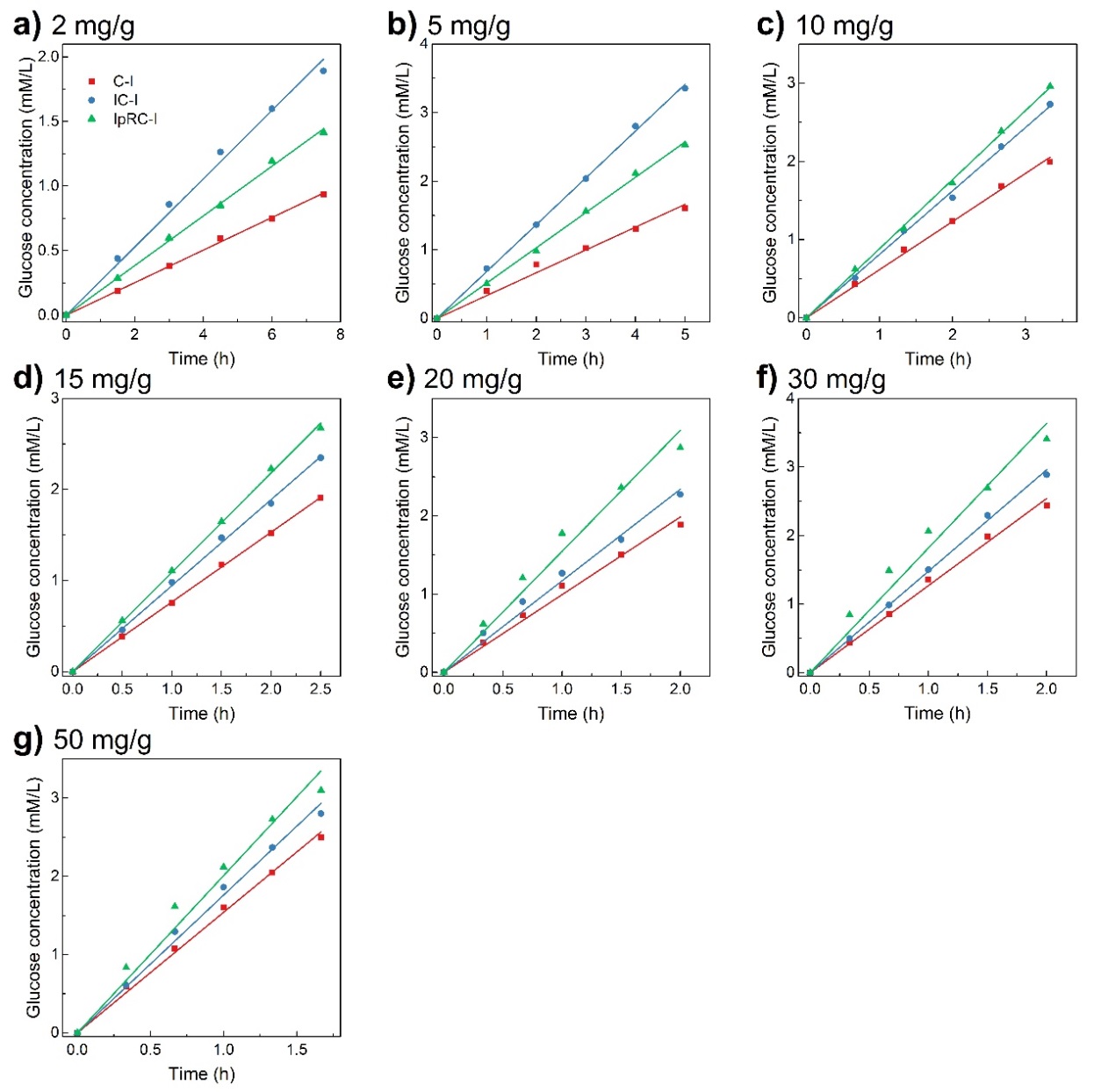


**Fig. S****2** Effect of enzyme loadings on the initial rate of glucose release during enzymatic hydrolysis. Conditions: cellulose substrate concentration 1%, 50 mM acetate acid buffer (pH 5.0), temperature 50 °C, cellulases complexes of 90% Cellulast 1.5L and 10% β-glucosidase. Turnover of frequency (TOF) can be calculated from these experimental data through the glucose concentration variance per unit time per unit enzyme.


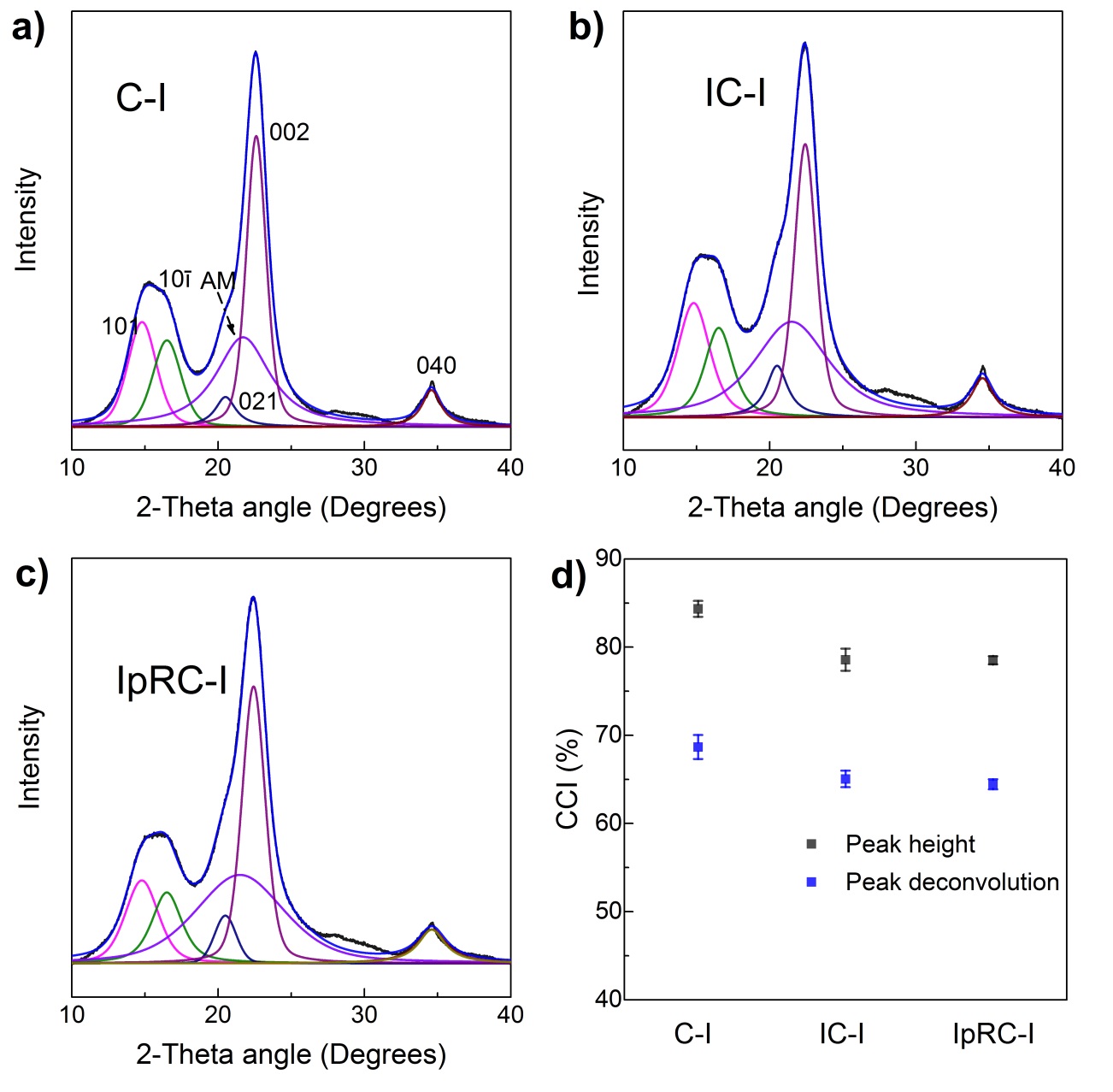


**Fig. S3** XRD of C-I, IC-I and IpRC-I cellulose substrates. Scans were obtained from 2è = 5 to 45, 30 min per sample. The cellulose crystallinity index was commonly estimated using two methods, XRD peak height and XRD peak deconvolution. Crl values calculated by peak height are always higher than these by peak deconvolution method.


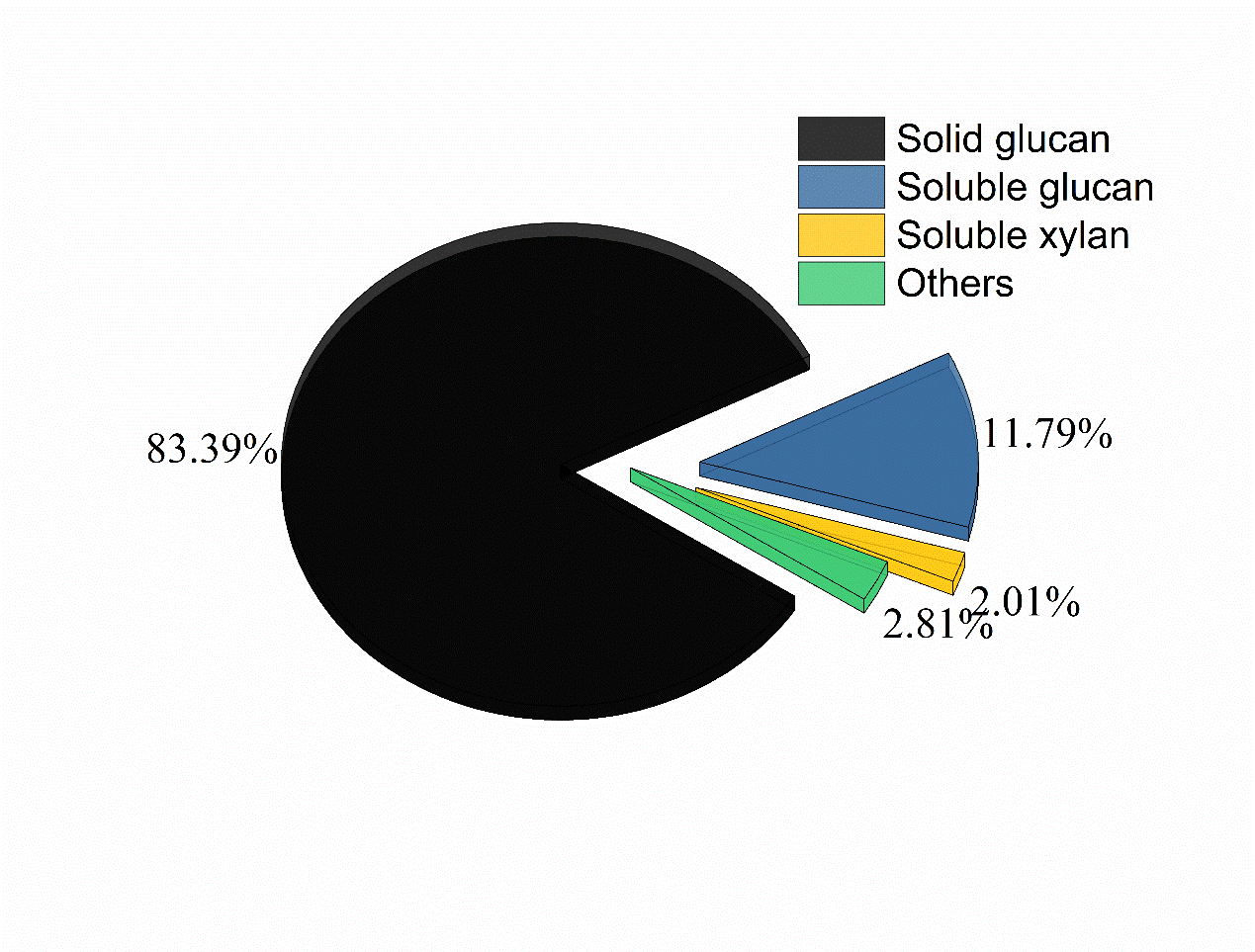


Fig. S4 Soluble oligosaccharides concentrations analyzed by HPLC method after Avicel cellulose PH-101 is irradiated at 600 KGy.


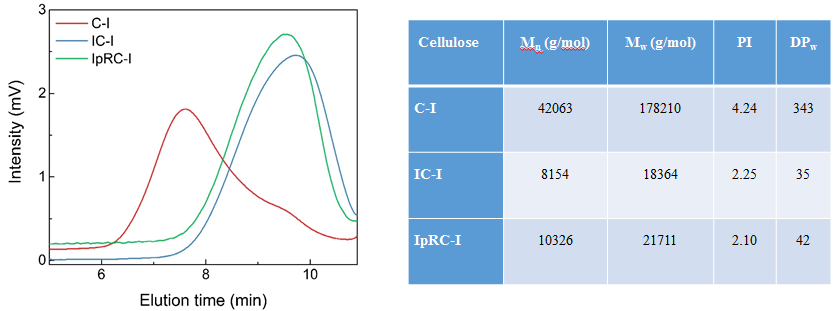


**Fig. S5** Molecular mass weight of cellulose substrates C-I, IC-I and IpRC-I analyzed by GPC. The conditions were: THF was used as eluent at the rate of 1.0 mL/min, injection volume was 50.0 ìL. Column temperature was 35 °C. The detected molecular weight ranges were 500 to 4×10^6^ Da. Degree of polymerization (DP) were calculated using these molecular weights dividing by 519, the molecular weight of cellulose tricarbanilate monomer. Mn means mass weight of number, g/mol; Mw is the molecular mass of weight, g/mol; PI is polydispersity index, which is calculated by Mn divided by Mw.


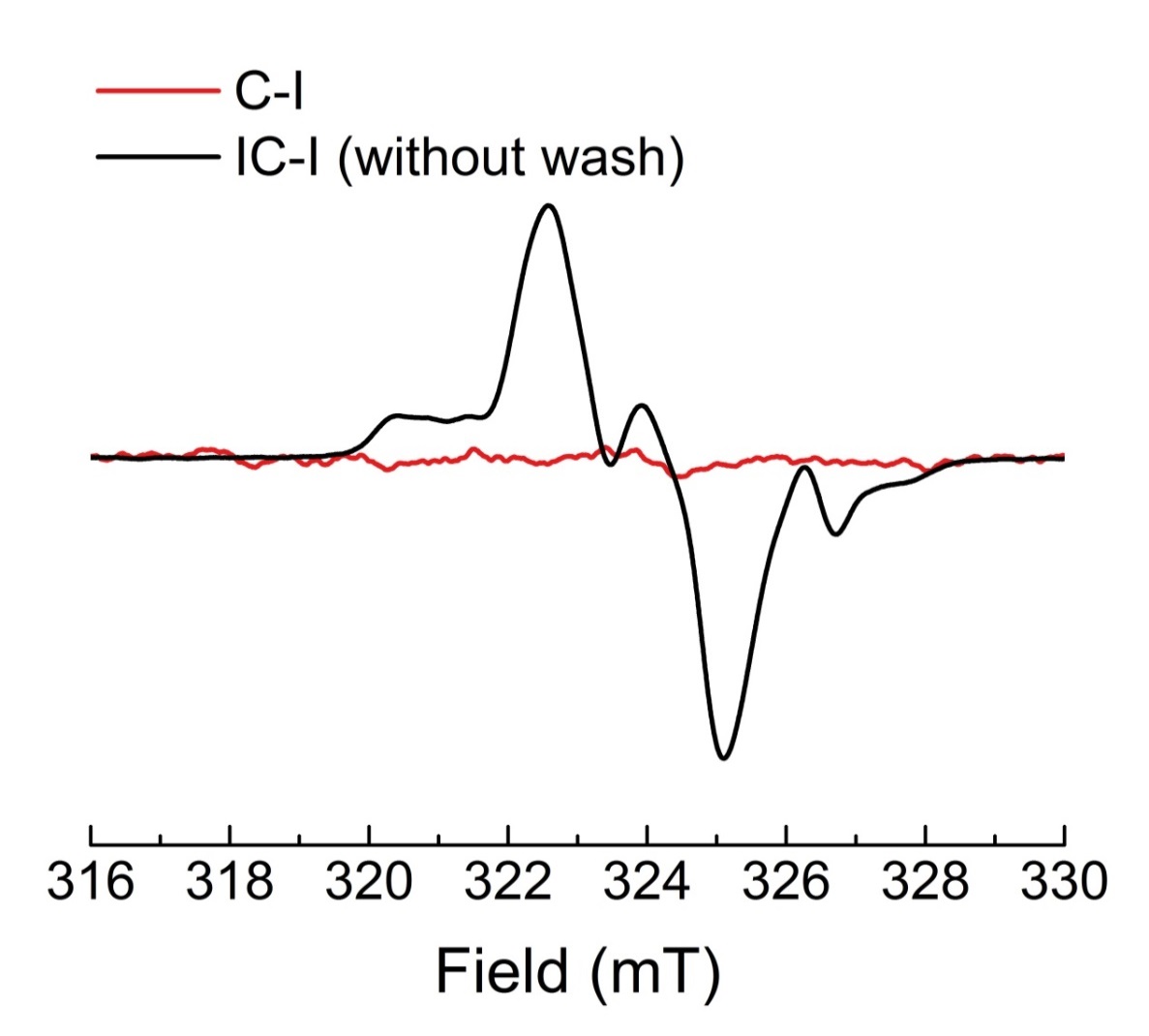


**Fig. S6** EPR of crystalline cellulose substrate before and after irradiation treatment. The EPR conditions were: microwave frequency (x-band) 9.06 GHz, microwave power 10 mW, center field 324 mT, sweep width 50 mT, modulation amplitude 0.35 mT, modulation frequency 100 KHz, sweep time 60 s, and temperature 37 °C.


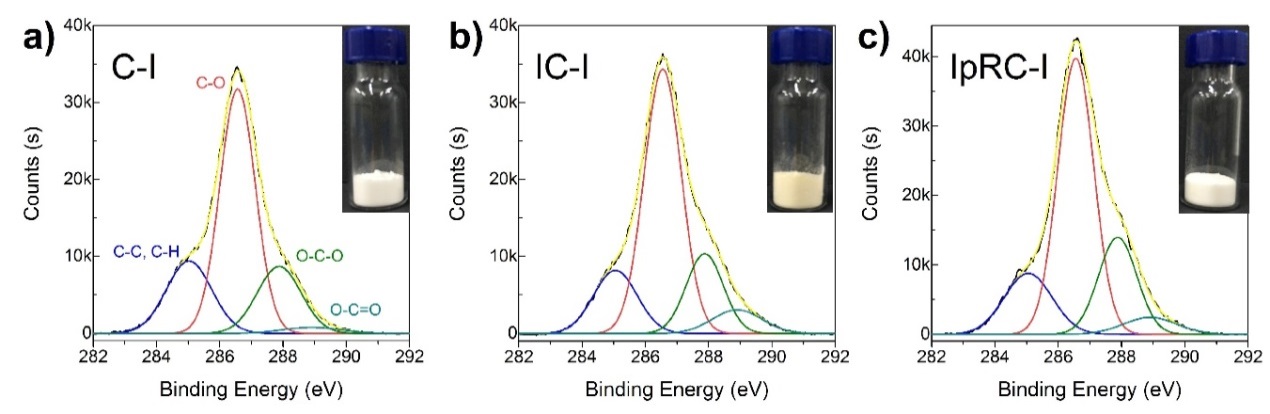


|  | **C** (%) | C1s composition (%) | | | | **O/C** ratio |
| --- | --- | --- | --- | --- | --- | --- |
|  |  | C1 | C2 | C3 | C4 |  |
| C-I | 59.5 | 21.56 | 56.96 | 19.27 | 2.21 | 0.67 |
| IC-I | 57.37 | 16.44 | 58.92 | 17.65 | 6.99 | 0.72 |
| IpRC-I | 58.62 | 16.96 | 56.99 | 21.22 | 4.83 | 0.69 |

**Fig. S7** XPS of C-I, IC-I and IpRC-I cellulose substrates. The analyzed area was 500 ìm×500 ìm. The carbon element signals were deconvoluted (within 0.2 eV) into C1 (284.8 eV), C2 (286.5 eV), C3 (287.9 eV) and C4 (288.8 eV) signals using XPS PEAK (Version 4.1).


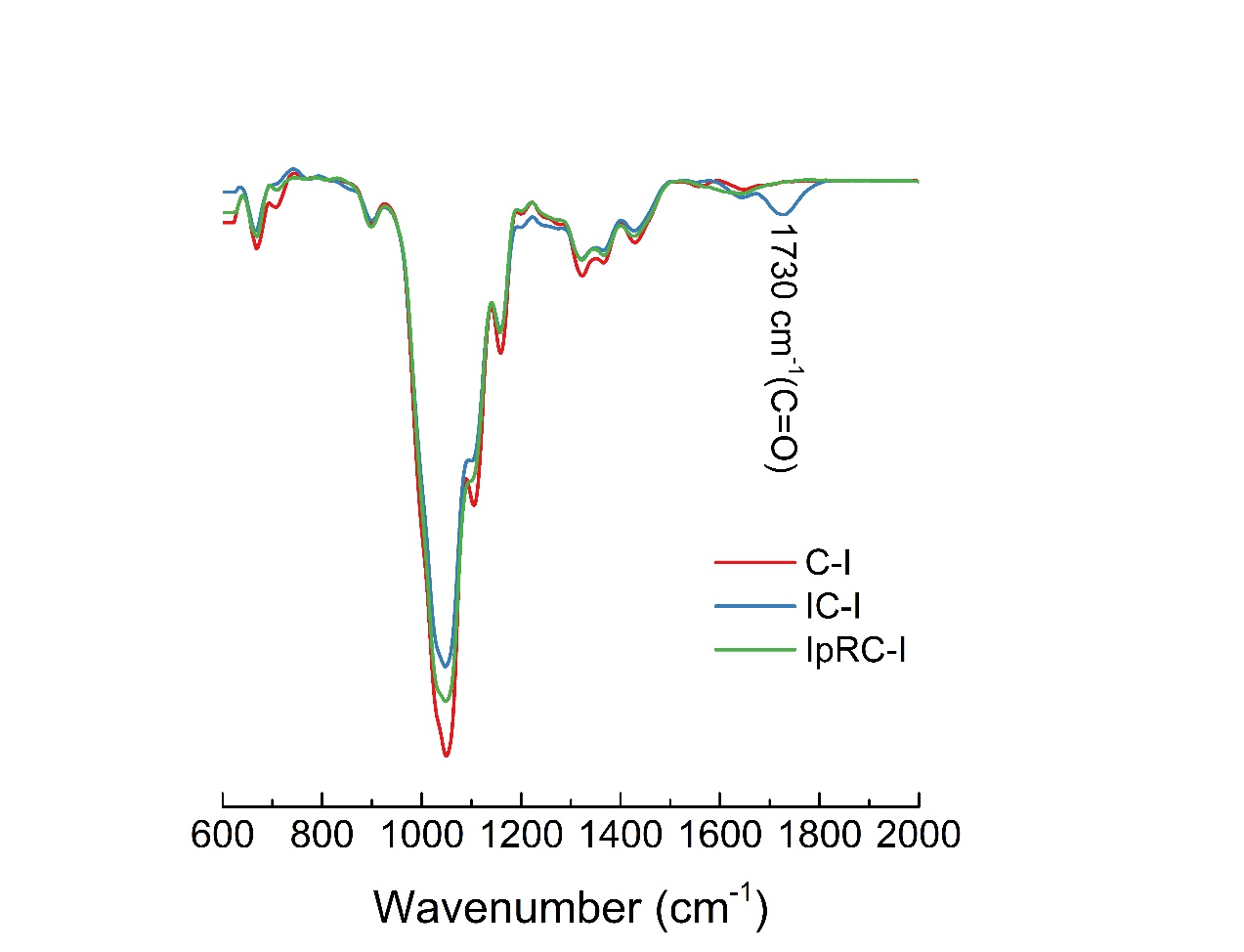


| Wavenumber, cm^-1^ | Function group | Assignment |
| --- | --- | --- |
| 800 – 950 | C-H | C-H deformation vibration in cellulose |
| ~1035 | C-O | C-O stretching vibration in cellulose |
| 1098/900 ^a^ |  | Amorphous to crystalline cellulose ratio |
| ~1637 | O-H | O-H bending vibration of adsorbed water molecules |
| ~1730 | C=O | Oxidation of cellulose |

**Fig. S8** ATR-FTIR of C-I, IC-I and IpRC-I cellulose substrates and the characteristic peaks assignment. The spectra were recorded from 600 to 4000 cm^-1^ at a resolution of 2 cm^-1^.


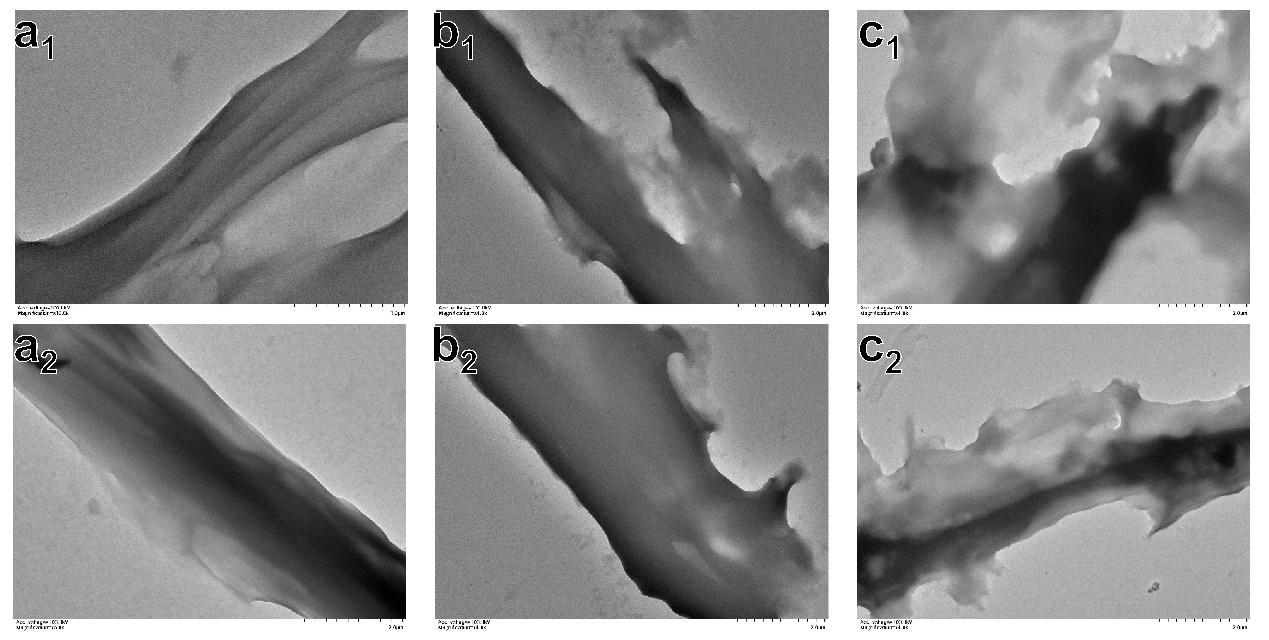


**Fig. S9** TEM micrographs of partially digested C-I, IC-I and IpRC-I cellulose particles. That cellulose was digested 24 h by cellulase from *T. reesei*. a_1_, a_2_ is digested C-I (42.5% conversion). b_1_, b_2_ is digested IC-I (66.2% conversion). c_1_, c_2_ is digested IpRC-I (81.6% conversion).
